# Probabilistic Deterministic Finite Automata and Recurrent Networks, Revisited

**DOI:** 10.3390/e24010090

**Published:** 2022-01-06

**Authors:** Sarah E. Marzen, James P. Crutchfield

**Affiliations:** 1 W. M. Keck Science Department, Pitzer, Scripps, and Claremont McKenna College, Claremont, CA 91711, USA; 2 Complexity Sciences Center, Physics Department, University of California at Davis, One Shields Avenue, Davis, CA 95616, USA

**Keywords:** time series prediction, finite state machines, hidden Markov models, recurrent neural networks, reservoir computers, long short-term memory

## Abstract

Reservoir computers (RCs) and recurrent neural networks (RNNs) can mimic any finite-state automaton in theory, and some workers demonstrated that this can hold in practice. We test the capability of generalized linear models, RCs, and Long Short-Term Memory (LSTM) RNN architectures to predict the stochastic processes generated by a large suite of probabilistic deterministic finite-state automata (PDFA) in the small-data limit according to two metrics: predictive accuracy and distance to a predictive rate-distortion curve. The latter provides a sense of whether or not the RNN is a lossy predictive feature extractor in the information-theoretic sense. PDFAs provide an excellent performance benchmark in that they can be systematically enumerated, the randomness and correlation structure of their generated processes are exactly known, and their optimal memory-limited predictors are easily computed. With less data than is needed to make a good prediction, LSTMs surprisingly lose at predictive accuracy, but win at lossy predictive feature extraction. These results highlight the utility of causal states in understanding the capabilities of RNNs to predict.

## 1. Introduction

Many real-world tasks rely on prediction. Given past stock prices, traders try to predict if a stock price will go up or down, adjusting investment strategies accordingly. Given past weather, farmers endeavor to predict future temperatures, rainfall, and humidity, adapting crop and pesticide choices. Manufacturers try to predict which goods will appeal most to consumers, adjusting raw materials purchases. Self-driving cars must predict the motion of other objects on and off the road. Furthermore, when it comes to biology, evidence suggests that organisms endeavor to predict their environment as a key survival strategy [1,2,3]. One simple metric often used to evaluate the quality of our predictive algorithms is simply the accuracy of our predictions—how well we can predict what will happen next given what has happened previously.

However, we also care about the cost of formulating and communicating a prediction of the next symbol in some sequence of symbols, either to another person or from one part an organism to another. Costs of formulation might include the time, memory, and/or energy taken to compute a prediction. Once the prediction is made, it is often communicated to some other downstream region that will use the prediction to take an action. This communication requires some amount of channel capacity, and channel capacity can be energetically expensive. All other concerns equal, one is inclined to employ a predictor with a lower transmission rate [4].

Here, we focus solely on communication, ignoring costs in formulating the prediction. As such, note that transmission rate is unrelated to sample complexity or time complexity. Rather, we allow for an unbounded number of samples in testing (thus avoiding the question of generalization error) and an unbounded time to train and compute predictions, and merely ask: what channel capacity do we need to faithfully communicate the predictions?

Simultaneously optimizing the objectives—high predictive accuracy and low code rate—leads to *predictive rate-distortion* [5,6,7]. The predictive rate-distortion curve separates combinations of achievable rates and distortions from unachievable rates and distortions. The closer a lossy predictive compressor is to the curve, the better. This diagnosis has been used, for example, to suggest that salamander retinal ganglion cells are near-optimal lossy predictors of visual input [8].

Surprisingly, we do not yet know how well recurrent neural networks perform relative to the predictive rate-distortion curve, though rate-distortion curves have been used to explain and calibrate the performance of artificial feedforward neural networks [9,10]. Note that recurrent neural networks allow us to store information, in principle, about semi-infinite pasts, while feedforward neural networks only allow for storage of finite pasts. The following calibrates the performance of various predictors (generalized linear models, reservoir computers, and recurrent neural networks) using the predictive rate-distortion curve. We stimulate predictors with output of probabilistic deterministic finite automata (PDFA), also called unifilar hidden Markov models in information theory [11]. The PDFAs used in the following are simple, in that their statistical complexity [12] and excess entropy [13,14] are finite and relatively small. The following explores PDFAs since optimal predictors of the time series they generate are easily computed [12], and the tradeoffs between code rate and predictive accuracy (encapsulated by the predictive rate-distortion function) are easily computed as well [7].

This work builds on seminal results establishing that both reservoir computers (RCs) [15,16] and recurrent neural networks (RNNs) [17] can reproduce any dynamical system, when given a sufficient number of nodes. Further work gave example RNNs that faithfully reproduce finite state automata, to the point that RNN nodes mimicked the automata states [18], and established bounds on the required RNN complexity [19]. One would conjecture, then, that Long Short-Term Memory (LSTM) architectures—an easily-trainable RNN variety [20,21]—should easily learn to predict the outputs of PDFAs. The further question we ask is: do these models not only predict, but predict *efficiently*?

We use predictive rate-distortion curves to calibrate the performance of three time series predictors: generalized linear models (GLMs) [22], RCs [15,16], and LSTMs [20]. Unsurprisingly, LSTMs are generally more efficient than reservoirs, which are generally more efficient than GLMs. Perhaps unsurprisingly, LSTMs are less accurate than both methods, seemingly due to overfitting. Surprisingly, despite the simplicity of the generated stochastic time series, we find that all tested prediction methods can fail to attain maximal predictive accuracy (measured by the probability of being correct) by as much as 50% and often need higher rates than necessary to attain maximal predictive performance. However, existing methods for inferring PDFAs [23] can correctly infer the PDFA and generate the optimal predictor with orders-of-magnitude less data. This leads us to conclude that prediction algorithms that first infer *causal states* [6,23,24,25] can surpass trained RNNs if the time series in question has (approximately) finite causal states, sometimes also called *predictive state representations* [26].

In Section 2, we describe how rate-distortion functions can provide a benchmark for prediction algorithms. In Section 3, we describe PDFAs, GLMs, RCs, and LSTMs. In Section 4, we describe our results. Section 5 summarizes our conclusions.

## 2. Rate-Distortion Benchmarks for Prediction Algorithms

**Figure 1:**
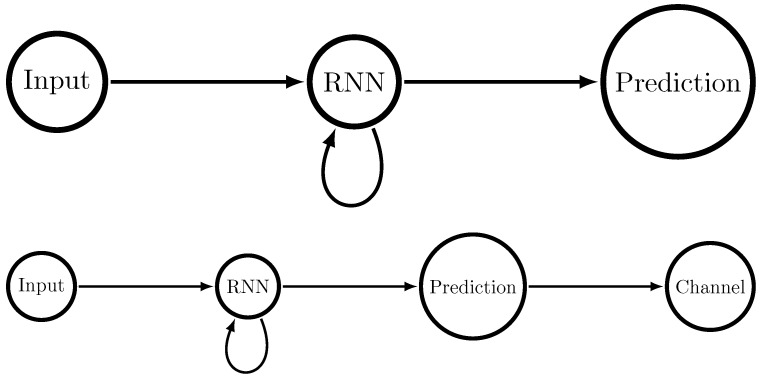
At (**top**), a typical setup for a recurrent neural network (or any other predictor): input is sent to the recurrent neural network, which makes a prediction about future inputs. At (**bottom**), our setup for a recurrent neural network in which predictions must be made and the prediction must be communicated losslessly through the channel.

Typically, when one talks about recurrent neural networks, one considers a setup as in Figure 1 (top). Input is sent to the network, which updates its state based on both the input and its previous state. The network’s state is then used to make a prediction. The only metric that characterizes the final performance of the network, post-training, is the prediction accuracy—how well it predicts future symbols given past symbols.

We now augment that setup slightly. Consider a channel over which the prediction must be communicated, as in Figure 1 (bottom). Now there are two metrics that characterize the network’s performance, post-training: the predictive accuracy and the required channel capacity. In the particular setup of Figure 1 (bottom), the required channel capacity must be at least the entropy of the predictions [4]. If one is allowed longer blocklengths, meaning that one can communicate several predictions at once using the channel, the required channel capacity somewhat diminishes.

**Figure 2:**
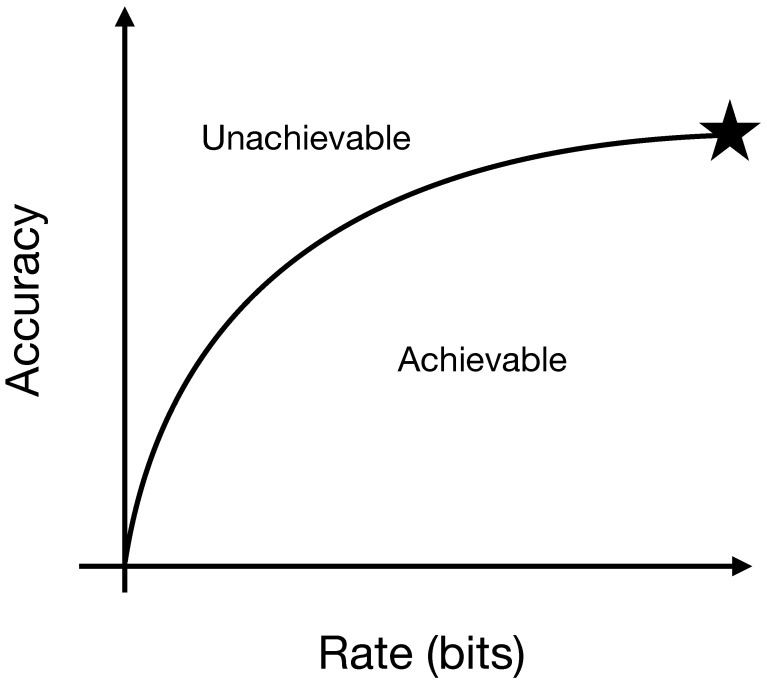
A sample predictive rate-accuracy curve, which is dependent not on how we process the time series but only on intrinsic properties of the time series. It is quite possible, and typical, to have zero rate and a nonzero predictive accuracy, and so the meeting of the *x*-axis and *y*-axis is not at the origin. The rate can run between zero and one bit for the binary-valued time series we study here. The starred point, which encodes the rate and accuracy of a minimal optimal predictor, has a rate of the single-symbol Shannon entropy of the time series and a predictive accuracy that depends in a complicated way on the specific time series. (Note the slight difference between this communication setup and that of standard predictive rate-distortion.) It is possible to have rates larger than the rate of the starred point, up to and including one bit.

One can now trace out a plane of the two metrics, prediction accuracy and channel capacity, and ask which combinations of the two are achievable. The curve that separates the achievable combinations from the unachievable combinations is called the predictive rate-accuracy curve, very closely related to the predictive rate-distortion curve. See Figure 2.

Let *R* be the random variable representing our representation of the past that we use to predict the future, and *r* be its realization. When the accuracy is the conditional mutual information $I[\vec{X};\overset{\leftarrow }{X}|R]$, the predictive rate-accuracy function is exactly the predictive information curve [5,6]. Finding representations that lie on the information curve motivates slow feature analysis [27], recovers canonical correlation analysis [28], and identifies the minimal sufficient statistics of prediction—the causal states [5]. Predictive information curves have even been used to evaluate the predictive efficiency of salamander retinal neural spiking patterns [8].

Here, however, we work only with binary processes, and we adopt the stance that predictive accuracy could be taken to be the probability that one’s prediction is correct. Accordingly, we force our representation r∈{0,1} to be a prediction, and calculate *accuracy* via:

$$\begin{array}{c} a\left(r_{t},x_{t+1}\right)=1-\delta_{r_{t},x_{t+1}}\;, \end{array}$$

which implies:

$$\begin{array}{c} E\left[a\right]=\sum_{{\overset{\leftarrow }{x}}_{t}}p\left({\overset{\leftarrow }{x}}_{t}\right)\sum_{r_{t}=x_{t+1}}p\left(r_{t}|{\overset{\leftarrow }{x}}_{t}\right)p\left(x_{t+1}|{\overset{\leftarrow }{x}}_{t}\right)\;. \end{array}$$

The choice of distortion or accuracy measure is an important one, and determined by one’s particular application.

There is another way to understand predictive rate-accuracy curves. With an eye to making contact with nonpredictive rate-distortion theory, we summarize the setup of predictive rate-accuracy as follows. Semi-infinite pasts are drawn independently from the same process-dependent distribution and sent to an encoder, which then produces a prediction or a probability distribution over possible predictions. A predictive distortion measures how far the estimated predictions differ from correct predictions. Distortion is often taken, for example, to be the Kullback-Leibler divergence between the true distribution $p(\vec{x}|\overset{\leftarrow }{x})$ over futures $\vec{x}$ conditioned on the past $\overset{\leftarrow }{x}$ and the distribution $p(\vec{x}|r)$ over futures conditioned on our *representation* *r* [29]. A predictive accuracy might then be some maximal achievable accuracy minus the predictive distortion. The predictive rate-accuracy curve R(A), the minimal necessary rate at a given expected accuracy, separates the plane of rates and predictive distortions into regions of achievable and unachievable combinations. A slight variant of the rate-distortion theorem gives:

(1) $$\begin{array}{c} R\left(A\right)=\min_{p\left(\vec{x}|r\right):E\left[a\right]\geq A}I\left[\overset{\leftarrow }{X};R\right]\;, \end{array}$$

where I[·;·] is the mutual information.

## 3. Background

In what follows, we review time-series generation and the widely-used prediction methods we compare. We first discuss PDFAs and then prediction methods.

### 3.1. PDFAs and Predictive Rate-Distortion

We focus on minimal PDFAs—for a given stochastic process, that with the smallest number of states. A PDFA consists of a set S of states σ∈S, a set A of emission symbols, and transition probabilities $p(\sigma_{t+1},x_{t}|\sigma_{t})$, where $\sigma_{t}$, $\sigma_{t+1}\in S$ and $x_{t}\in A$. The “deterministic” descriptor comes from the fact that $p(\sigma_{t+1}|x_{t},\sigma_{t})$ has support on only one state. (This is “determinism” in the sense of formal language theory [30]—an automaton deterministically *recognizes* a string—not in the sense of nonstochastic. It was originally called *unifilarity* in the information theoretic analysis of hidden Markov chains [11]. Thus, PDFAs are also known as *unifilar hidden Markov models* [12].)

Here, we concern ourselves with minimal and binary-alphabet (A={0,1}) PDFAs. In dynamical systems theory minimal unifilar HMMs (minimal PDFAs) are called *ϵ-machines* and their states σ *causal states*. Due to the automaton’s determinism, one can uniquely determine the state from the past symbols with probability 1. Each state is therefore a cluster of pasts that have the same conditional probability distribution over futures. As a result, all that one needs to know to optimally predict the future is given by the causal state [12].

**Figure 3:**
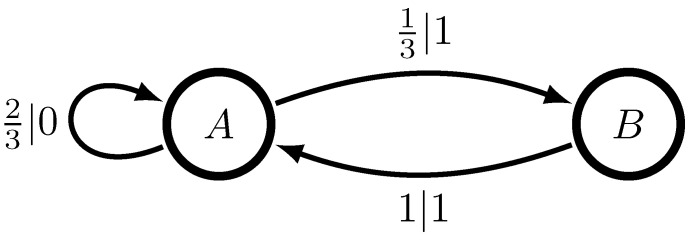
Minimal two-state PDFA that generates the Even Process, so-called since there are always an even number of 1s between 0’s. Arrows indicate allowed transitions, while transition labels p|s indicate the transition (and so too emission) probabilities p∈[0,1] for the symbol s∈A. Given a current state and next symbol, one knows the next state—the deterministic or unifilar property of this PDFA.

For example, the simple two-state PDFA shown in Figure 3 generates the Even Process: only an even number of 1’s are seen between two successive 0’s. This leads to a simple prediction algorithm: find the parity of the number of 1’s since the last 0; if even, we are in state *A*, so predict 0 and 1 with equal probability; if odd, we are in state *B*, so predict 1. There is only one past for which our prediction algorithm yields no fruit: given the past of all 1s a single state is never identified. One only knows that the machine is in either state *A* or *B* and the best prediction is a mixture of what the states indicate. Even though that past occurs with probability 0, it causes the Even Process to be an infinite-order Markov Process [31]. See Ref. [32] for a measure-theoretic treatment.

Causal states and ϵ-machines can be inferred from data in a variety of ways [6,23,25,33].

The causal states are uniquely useful to calculating predictive rate-distortion curves. Under weak assumptions, the predictive rate-accuracy function of Section 2 becomes:

$$\begin{array}{c} R\left(A\right)=\min_{p(r|\sigma ):E[d]\geq A}I\left[S;R\right] \end{array}$$

with:

$$\begin{array}{c} E\left[d\right]=\sum_{\sigma_{t}}p\left(\sigma_{t}\right)\sum_{x_{t+1}=r_{t}}p\left(r_{t}|\sigma_{t}\right)p\left(x_{t+1}|\sigma_{t}\right)\;. \end{array}$$

See Ref. [7] for the proof. With this substitution—of a finite object (S) for an infinite one ($\overset{\leftarrow }{X}$)—the Blahut-Arimoto algorithm can be used to accurately calculate the predictive rate-accuracy function, in that the algorithm provably converges to the optimal p(r|σ) [34]. The same cannot be said of the predictive information curve [7], which converges to a local optimum of the objective function, but may not converge to a global optimum.

In practice, we always augment the predictive rate-accuracy function with the rate and accuracy of the optimal predictor, which is (as described earlier) straightforwardly derived from the ϵ-machine. Simply put, we infer the causal state $\sigma_{t}$ from past data and predict the next symbol to be $\arg\max_{x_{t+1}}p\left(x_{t+1}|\sigma_{t}\right)$.

The following tests the various time series predictors on all of the (uniformly sampled) binary-alphabet ϵ-machine topologies [35] with randomly-chosen emission probabilities. Due to the super-exponential explosion of the set of topological ϵ-machines with number of states, we only look at binary-alphabet machines with four or fewer (causal) states. (There are 1338 unique topologies for four states, but over $10^{6}$ for six states.) The analysis discards any ϵ-machine with zero-rate optimal predictor, which can arise depending on the emission probabilities.

### 3.2. Time Series Methods

We focus on three methods for time series prediction: generalized linear models (GLM), reservoir computers (RCs), and LSTMs.

The GLM we use predicts $x_{t}$ from a linear combination of the last *k* symbols $x_{t-k},x_{t-k+1},$$\ldots ,x_{t-1}$. More precisely, a GLM models the probability of $x_{t}$ being a 0 via:

(2) $$\begin{array}{c} p_{GLM}\left(x_{t}=0|x_{t-k},\ldots ,x_{t-1}\right)=\frac{e^{w_{k}x_{t-k}+\ldots +w_{1}x_{t-1}+w_{0}}}{1+e^{w_{k}x_{t-k}+\ldots +w_{1}x_{t-1}+w_{0}}}\;. \end{array}$$

The model’s estimate of the probability of $x_{t}=1$ follows:

(3) $$p_{GLM}\left(x_{t}=1|x_{t-k},\ldots ,x_{t-1}\right)=\frac{1}{1+e^{w_{k}x_{t-k}+\ldots +w_{1}x_{t-1}+w_{0}}}\;.$$

We use Scikit-learn logistic regression to find the best weights $w_{0},w_{1},\ldots ,w_{k}$. Predictions are then made via $\arg\max_{x_{t}}p_{GLM}\left(x_{t}|x_{t-k},\ldots ,x_{t-1}\right)$.

The RC is more powerful in that it uses logistic regression with features that contain information about symbols arbitrarily far into the past. We employ a tanh activation function, so that the reservoir’s state advances via:

(4) $$\begin{array}{c} h_{t+1}=\tanh\left(Wh_{t}+vx_{t}+b\right) \end{array}$$

and initialize W,v,b with i.i.d. normally distributed elements. The matrix *W* is then scaled so that it is near the “edge of chaos” [36,37,38,39], where RCs are conjectured to have maximal memory [40,41]. We then use logistic regression with $h_{t}$ as features to predict $x_{t}$:

$$\begin{array}{cc} p_{reservoir}\left(x_{t}=0|h_{t}\right) & =\frac{e^{w^{⊤}h_{t}+w_{0}}}{1+e^{w^{⊤}h_{t}+w_{0}}}\;,\\ p_{reservoir}\left(x_{t}=1|h_{t}\right) & =\frac{1}{1+e^{w^{⊤}h_{t}+w_{0}}}\;. \end{array}$$

It is straightforward to devise a weight matrix *W* and bias *b* so that $p_{reservoir}\left(x_{t}|h_{t}\right)$ attains the restricted linear form of $p_{GLM}$ of Equations (2) and (3). That is, RCs are more powerful than GLMs, as they use nonlinear functions of semi-infinite pasts for their summary statistics. We use Scikit-learn logistic regression to find the best weights $w_{0}$ and *w*. Note that the weights *W*, *v*, and *b* are not learned, but held constant; we only train *w* and $w_{0}$. Predictions are made via $\arg\max_{x_{t}}p_{reservoir}\left(x_{t}|h_{t}\right)$.

Finally, we analyze the LSTM’s predictive capabilities. LSTMs are no more powerful than vanilla RNNs; e.g., those as in Equation (4). However, they are far more trainable in that it is possible to achieve good results without extensive hyperparameter tuning [21]. An LSTM has several hidden states $f_{t}$, $i_{t}$, $o_{t}$, $c_{t}$, and $h_{t}$ that update via the following:

$$\begin{array}{cc} f_{t} & =\sigma_{g}\left(W_{f}x_{t}+U_{f}h_{t-1}+b_{f}\right)\\ i_{t} & =\sigma_{g}\left(W_{i}x_{t}+U_{i}h_{t-1}+b_{i}\right)\\ o_{t} & =\sigma_{g}\left(W_{o}x_{t}+U_{o}h_{t-1}+b_{o}\right)\\ c_{t} & =f_{t}⊙c_{t-1}+i_{t}⊙\sigma_{c}\left(W_{c}x_{t}+U_{c}h_{t-1}+b_{c}\right)\\ h_{t} & =o_{t}⊙c_{t}\;, \end{array}$$

where $\sigma_{g}$ is the sigmoid function and $\sigma_{c}$ is the hyperbolic tangent. The variable $c_{t}$ is updated linearly, therefore avoiding issues with vanishing gradients [42]. Meanwhile, the gating function $f_{t}$ allows us to forget the past selectively. We then predict the probability of $x_{t}$ given the past using:

(5) $$\begin{array}{cc} p_{LSTM}\left(x_{t}=0|h_{t}\right) & =\frac{e^{w^{⊤}h_{t}+w_{0}}}{1+e^{w^{⊤}h_{t}+w_{0}}}\;,\\ p_{LSTM}\left(x_{t}=1|h_{t}\right) & =\frac{1}{1+e^{w^{⊤}h_{t}+w_{0}}}\;. \end{array}$$

Weights *w* and $w_{0}$ are learned while we estimate parameters $W_{f}$, $U_{f}$, $b_{f}$, $W_{i}$, $U_{i}$, $W_{o}$, $U_{o}$, $b_{o}$, $W_{c}$, $U_{c}$, and $b_{c}$ to maximize the log-likelihood. Predictions are made via $\arg\max_{x_{t}}p_{LSTM}\left(x_{t}|h_{t}\right)$.

Predictive accuracy is calculated by comparing the predictions to the actual values of the next symbol and counting the frequency of correct predictions. The code rate is calculated via the prediction entropy [4].

## 4. Results

An aim here is to thoroughly and systematically analyze the predictive accuracy as measured by the probability of correctly guessing the next symbol and code rate of our three time series predictors of a large swath of PDFAs in the small-data limit, in which only 5000 samples are shown to the RNN. To implement this, we ran through Ref. [35]’s topological ϵ-machine library—binary-alphabet PDFAs with four states or less and randomly chosen emission probabilities, in which transition probabilities were drawn from a uniform distribution. For each PDFA, we generated a length-5000 time series. The first half was presented to a predictor and used to train its weights. We then evaluated each time series predictor based on its predictions for the second half of the time series. Predictive accuracy and code rate were calculated and compared to the predictive rate-distortion function. Predictive accuracy was calculated as the probability of having a correct prediction; code rate was calculated empirically as the single-symbol entropy of the predictions [14].

Note that Bayesian structural inference (BSI) provides a useful comparison [23]. In BSI, we compute the maximum a posteriori (MAP) estimate of the PDFA generating an observed time series, and use this MAP estimate to build an optimal predictor of the process. BSI can correctly infer the PDFA essentially 100% of the time with orders-of-magnitude less data than used to monitor the three prediction methods tested here. Hence, it achieves optimal predictive accuracy with minimal rate. Our aim is to test the ability of GLMs, RCs, and RNNs to equal BSI’s previously-published performance.

The time series predictors used have hyperparameters. A variety of orders (*k*’s) were used for the GLMs and reservoirs and LSTMs of different sizes (number of nodes) were tested. Learning rate and optimizer type, including gradient descent and Adam [43], were also varied for the LSTM, with little effect on results. Regularization was necessary and utilized in both $L_{1}$ and $L_{2}$ forms on all three predictors. As is typical, a validation set was used to select the strength and type of regularization, and results were reported on a separate test set. In total, 5000 steps of the time series were simulated, which was small enough to test how these machine learning methods responded to too little data, but enough data that the machine learning methods could have picked up on patterns.

### 4.1. The Difference between Theory and Practice: The Even and Neven Process

We first analyze two easily-described PDFAs, deriving RNNs that correctly infer causal states and, therefore, that match the optimal predictor—the ϵ-machine. We then compare the trained GLMs, RCs, and LSTMs to the easily-inferred optimal predictors. In theory, RCs and LSTMs should be able to mimic the derived RNNs, in that it is possible to find weights of an RC and LSTM that yield nodes that mimic the causal states of the PDFA. In practice, surprisingly, RCs and LSTMs have some difficulty.

First, we analyze the Even Process shown in Figure 3. The optimal prediction algorithm is easily seen by inspection of Figure 3. When we determine the machine is in state *A*, we predict a 0 or a 1 with equal probability; if it is in state *B*, we predict a 1. We determine whether or not it is in state *A* or *B* by the parity of the number of 1s since the last 0. If odd, it is in state *B*; if even, it is in state *A*. The inferred state is easily encoded by the following RNN:

(6) $$h_{t+1}=x_{t}\left(1-h_{t}\right)\;.$$

If $x_{t}$ is 0, the hidden state of the RNN “resets” to 0; e.g., state *A*. If $x_{t}=1$, then the hidden state updates by flipping from 0 to 1 or vice versa, mimicking the transitions from *A* to *B* and back. One can show that a one-node LSTM hidden state $h_{t}$ can, with proper weight choices, mimic the hidden state of Equation (6). With the correct hidden state inferred, it is straightforward to find *w* and $w_{0}$ such that Equation (5) yields optimal (and correct) predictions.

**Figure 4:**
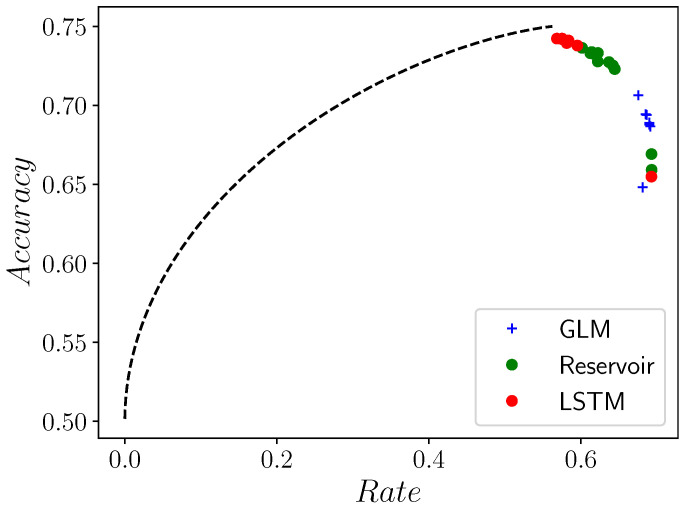
Predictive rate–accuracy curve for the Even Process in Figure 3, along with empirical predictive accuracies and rates of GLMs, RCs, and LSTMs of various sizes: orders range from 1–10 for GLMs, number of nodes range from 1–61 for RCs, and number of nodes range from 1–121 for LSTMs. Despite the Even Process’ simplicity, there is a noticeable difference between the predictors’ performances and between their performances and the optimal achievable performance.

As one might then expect, and as Figure 4 confirms, LSTMs tend to have rates that are close to the optimal (maximal) rate and predictive accuracies that are only slightly below the optimal predictive accuracy. RCs and GLMs tend to have higher rates and lower predictive accuracies, but they are still within ∼13% of optimal. We can see this qualitatively just by examining the predictive rate-accuracy curve in Figure 4: the closer that a point is to the curve, the more efficiently that predictor predicts. Among the points on the curve, potentially the most desirable point is the one at the highest achievable accuracy, at the top right. The points from the LSTMs tend to be closer to the curve and closer to the point at the top right, followed by RCs, and followed by GLMs. Interestingly, the points from all processes lie on a one-dimensional curve, speaking to some hidden simplicity in the relationship between rate and accuracy that likely holds only for binary-valued processes.

As one might also expect, LSTMs and RCs with additional nodes and GLMs with higher orders (higher *k*) have higher predictive accuracies than LSTMs and RCs with fewer nodes and GLMs with lower orders. However, viewed another way, given the simplicity of the stimulus—indeed, given that a one-node LSTM can, in theory, learn the Even Process—the gap from the predictors’ rates and accuracies to the optimal combinations of rate and accuracy is surprising. It is also surprising that none of the three predictors’ rates fall below the maximal optimal rate.

**Figure 5:**
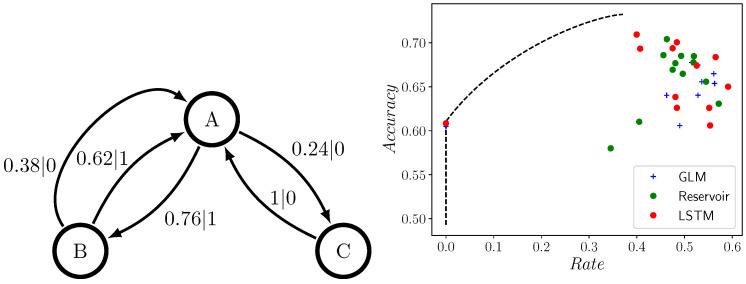
Predictive rate-accuracy curve for the Neven Process (PDFA shown at left), along with empirical predictive accuracies and rates of GLMs, RCs, and LSTMs of various sizes: orders range from 1–10 for GLMs, number of nodes range from 1–61 for RCs, and number of nodes range from 1–121 for LSTMs. Despite Neven Process’ simplicity, there is a noticeable gap between the predictor’s performance and the optimal performance achievable.

Figure 5 introduces a similarly-simple three-state PDFA. If a 1 is observed after a 0, we are certain the machine is in state *B*; after state *B*, we know it will transition to state *A*; and then the parity of 0s following transition to state *A* tells us if it is in state *A* (even) or state *B* (odd). This PDFA is a combination of a Noisy Period-2 Process (between states *A* and *B*) and an Even Process (between states *A* and *C*).

Given the Neven Process’s simplicity, it is unsurprising that we can concoct an RNN that can infer the internal state. Let $h_{t}=\left(h_{t,A},h_{t,B},h_{t,C}\right)$ be the hidden state that is (1,0,0) if the internal state is *A*, (0,1,0) if the internal state is *B*, and (0,0,1) if the internal state is *C*. By inspection, we have:

$$\begin{array}{cc} h_{t+1,A} & =1-h_{t,A}\\ h_{t+1,B} & =x_{t}h_{t,A}\\ h_{t+1,C} & =\left(1-x_{t}\right)h_{t,A}\;. \end{array}$$

One can straightforwardly find weights that lead to $p_{LSTM}\left(x_{t+1}|h_{t}\right)$ accurately reflecting the transmission (emission) probabilities. In other words, in theory a three-node RNN (and an equivalent three-node LSTM) can learn to predict the Neven process optimally.

However, the Neven Process’ simplicity is belied by the gap between the predictors’ accuracy and rate and the predictive rate-accuracy curve. In Figure 5, the point at zero rate implies that the predictor is spitting out the same symbol, regardless of input. The worst predictive accuracy falls short of the optimal by ∼15%, and none of the GLMs, RCs, or LSTMs get closer than ∼97% to optimal. Furthermore, almost all the rates surpass the maximal optimal predictor rate.

### 4.2. Comparing GLMs, RCs, and LSTMs

We now analyze the combined results obtained over all minimal PDFAs up to four states using two metrics. (Again, recall that they are 1338 unique machine topologies.) To compare across PDFAs, we first normalize the rate and accuracy by the rate and accuracy of the optimal predictor. Then, we find the distance from the predictor’s rate and accuracy to the predictive rate-accuracy curve, which is similar in spirit to the metric of Ref. [44] and to the spirit of Ref. [8]. Note that this metric would have been markedly harder to estimate had we used nondeterministic probabilistic finite automata; that is, those without determinism (unifiliarity) in their transition structure [7].

**Figure 6:**
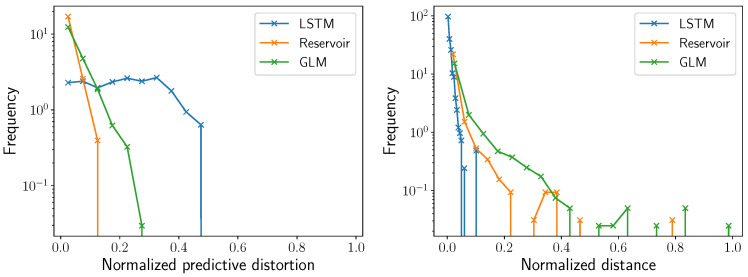
(**Left**) Histogram of normalized predictive distortions for LSTMs (blue), RCs (orange), and GLMs (green) using 798 distinct PDFAs. While LSTMs tend to have far higher predictive accuracies, they also have a much larger probability than reservoirs or GLMs do of having noticeable inaccuracies. Some recorded normalized predictive distortions were negative, indicating the effects of finite sample size. (**Right**) Histogram of normalized distances to the predictive rate-accuracy curve for LSTMs (blue), RCs (orange), and GLMs (green) using 798 distinct PDFAs. It is apparent that LSTMs are closer to the predictive rate-accuracy curves than reservoirs and GLMs.

Figure 6 showcases a histogram of the normalized distance to the predictive rate-accuracy curve, ignoring PDFAs for which the maximal optimal rate is 0 nats. The normalized distance for all three predictor types tends to be quite small, but even so, we can see differences in the three predictor types. LSTMs tend to have smaller normalized distances than RCs, and RCs tend to have smaller normalized distances to the predictive rate-accuracy curve than GLMs. In fact, LSTMs seem to be uniformly better lossy predictive feature extractors. Trained LSTMs on average have 0.8% normalized distance; RCs on average have 2.0% normalized distance; and GLMs on average have 4.5% normalized distance. When looking only at optimized LSTMs, RCs, and GLMs—meaning that the number of nodes or the order is chosen to minimize normalized predictive distortion—a few PDFAs still have high normalized predictive distortions of 4.6% for LSTMs, 9.7% for RCs, and 27.3% for GLMs.

The same trend holds for the percentage difference between the predictive accuracy and the maximal predictive accuracy, which we call the *normalized predictive distortion*, with a crucial modification. Trained LSTMs on average have 21.5% normalized predictive distortion; RCs on average have 1.8% normalized predictive distortion; and GLMs on average have 4.2% normalized predictive distortion. When looking only at optimized LSTMs, RCs, and GLMs—meaning that the number of nodes or the order is chosen to minimize normalized predictive distortion—a few PDFAs still have high normalized predictive distortions of 50% for LSTMs, 13.5% for RCs, and 25.5% for GLMs. However, perhaps the most interesting aspect of the Figure 6 is that LSTMs are far more likely than reservoirs or GLMs to have large normalized predictive distortions, surprisingly.

Unsurprisingly, increasing the GLM order and the number of nodes of the RCs and LSTMs tends to increase predictive accuracy and decrease the normalized distance.

Our final aim is to understand the PDFA characteristics that cause them to be harder to predict accurately and/or efficiently. We have two suspects, which are the most natural measures of process “complexity”. This first is the generated process’ entropy rate $h_{\mu }$, the entropy of the next symbol conditioned on all previous symbols, which quantifies the intrinsic randomness of the stimulus. The second is the generated process’ statistical complexity $C_{\mu }$, the entropy of the causal states, which quantifies the intrinsic memory in the stimulus. The more random a stimulus, the harder it would be to predict; imagine having to find the optimal predictor for a biased coin whose bias is quite close to 1/2. The more memory in a stimulus, the more nodes in a network or the higher the order of the GLM required, it would seem. We performed a multivariate linear regression, trying to use $h_{\mu }$ and $C_{\mu }$ to predict the minimal normalized predictive distortion and minimal normalized distance. We find a small and positive correlation for LSTMs, reservoirs, and GLMs for predicting minimal deviations in accuracy from perfection, with an $R^{2}$ of 0.189, 0.134, and 0.132, respectively. For all three types of prediction algorithms, statistical complexity $C_{\mu }$ is positively correlated with deviations in accuracy. Entropy rate is positively correlated with deviations in accuracy for GLMs and reservoirs but, surprisingly, not LSTMs. Interestingly, the performance GLMs and RCs is impacted by increased randomness and increased memory in the stimulus, while the LSTMs’ accuracy has little correlation with entropy rate and statistical complexity.

For the most part, we find that all three prediction methods–GLMs, RCs, and LSTMs—tend to learn to predict the PDFA outputs near-optimally, in that prediction accuracies differ from the optimal prediction accuracy by an average of roughly 5%. LSTMs outperform RCs, which outperform GLMs. However, we discovered simple PDFAs that cause the best LSTM to fail by as much as 5%, the best RC to fail by as much as 10%, and the best GLM to fail by as much as 27%.

Since none of the RNNs achieved perfect prediction accuracy, but the BSI method did [23], we conclude that existing methods for inferring causal states [6,23,25,33] are useful, despite the historically dominant reliance on RNNs. For example, as previously mentioned, Bayesian structural inference correctly infers the correct PDFAs almost 100% of the time, leading to essentially zero prediction error, on training sets that are orders of magnitude smaller than those used here [23].

## 5. Conclusions

We have known for a long time that reservoirs and RNNs can reproduce any dynamical system [15,16,17], and we have explicit examples of RNNs learning to infer the hidden states of a PDFA when shown the PDFA’s output [18]. We revisited these examples to better understand if the finding of Ref. [18] is typical. How often do RNNs and RCs learn efficient and accurate predictors of PDFAs, especially given that BSI can yield an optimal predictor with orders-of-magnitude less training data?

We conducted a rather comprehensive search, analyzing 798 randomly-generated PDFAs with four states or less. For each PDFA, we trained GLMs, RCs, and RNNs of varying orders or varying numbers of nodes. Larger orders and larger numbers of nodes led to more accurate and more efficient predictors. On average, the various time series predictors have ∼5% predictive distortion. In other words, we are apparently better at classifying MNIST digits than sometimes predicting the output of a simple PDFA. Again, existing algorithms [23] can optimally predict the output of the PDFAs considered here with orders-of-magnitude less training data. (MNIST is a database of handwritten digits.) These findings lead us to conclude that algorithms that explicitly focus on inference of causal states [6,23,24,25] have a place in the currently RNN-dominated field of time series prediction.

More importantly, in this small data limit, overfitting is an issue for LSTMs but not RCs or GLMs. However, LSTMs are somehow excellent lossy predictive feature extractors nonetheless. The mechanism behind this is a subject for future research.

Perhaps most importantly, the predictive rate-accuracy framework that we introduce here or similar such frameworks could be useful for calibrating the performance of time series predictors. We have added a cost that comparatively little research has focused on: that of communicating the prediction. Implicitly, we are arguing that predictors which do not have maximal predictive accuracy but do have small communication costs might be useful nonetheless.

## Data Availability

Available upon reasonable request from the authors.
